# Altered lung physiology in two cohorts after COVID-19 infection as assessed by computed cardiopulmonography

**DOI:** 10.1152/japplphysiol.00436.2022

**Published:** 2022-09-29

**Authors:** Snapper R. M. Magor-Elliott, Asma Alamoudi, Rebecca R. Chamley, Haopeng Xu, Tishan Wellalagodage, Rory P. McDonald, David O’Brien, Jonathan Collins, Ben Coombs, James Winchester, Ed Sellon, Cheng Xie, Dominic Sandhu, Christopher J. Fullerton, John H. Couper, Nicholas M. J. Smith, Graham Richmond, Mark P. Cassar, Betty Raman, Nick P. Talbot, Alexander N. Bennett, Edward D. Nicol, Grant A. D. Ritchie, Nayia Petousi, David A. Holdsworth, Peter A. Robbins

**Affiliations:** ^1^Department of Physiology, Anatomy and Genetics, University of Oxford, Oxford, United Kingdom; ^2^Oxford Centre for Clinical Magnetic Resonance Research, University of Oxford, Oxford, United Kingdom; ^3^Royal Centre for Defence Medicine, Birmingham, United Kingdom; ^4^Nuffield Department of Medicine, University of Oxford, Oxford, United Kingdom; ^5^Oxford University Hospitals NHS Foundation Trust, Oxford, United Kingdom; ^6^Department of Chemistry, University of Oxford, Oxford, United Kingdom; ^7^Division of Cardiovascular Medicine, Radcliffe Department of Medicine, University of Oxford, Oxford, United Kingdom; ^8^Academic Department of Medical Rehabilitation, Stanford Hall, Loughborough, United Kingdom; ^9^Royal Brompton Hospital, London, United Kingdom

**Keywords:** COVID-19, human, laser absorption spectroscopy, lung volumes, respiratory dead space

## Abstract

The longer-term effects of COVID-19 on lung physiology remain poorly understood. Here, a new technique, computed cardiopulmonography (CCP), was used to study two COVID-19 cohorts (MCOVID and C-MORE-LP) at both ∼6 and ∼12 mo after infection. CCP is comprised of two components. The first is collection of highly precise, highly time-resolved measurements of gas exchange with a purpose-built molecular flow sensor based around laser absorption spectroscopy. The second component is estimation of physiological parameters by fitting a cardiopulmonary model to the data set. The measurement protocol involved 7 min of breathing air followed by 5 min of breathing pure O_2_. One hundred seventy-eight participants were studied, with 97 returning for a repeat assessment. One hundred twenty-six arterial blood gas samples were drawn from MCOVID participants. For participants who had required intensive care and/or invasive mechanical ventilation, there was a significant increase in anatomical dead space of ∼30 mL and a significant increase in alveolar-to-arterial Po_2_ gradient of ∼0.9 kPa relative to control participants. Those who had been hospitalized had reductions in functional residual capacity of ∼15%. Irrespectively of COVID-19 severity, participants who had had COVID-19 demonstrated a modest increase in ventilation inhomogeneity, broadly equivalent to that associated with 15 yr of aging. This study illustrates the capability of CCP to study aspects of lung function not so easily addressed through standard clinical lung function tests. However, without measurements before infection, it is not possible to conclude whether the findings relate to the effects of COVID-19 or whether they constitute risk factors for more serious disease.

**NEW & NOTEWORTHY** This study used a novel technique, computed cardiopulmonography, to study the lungs of patients who have had COVID-19. Depending on severity of infection, there were increases in anatomical dead space, reductions in absolute lung volumes, and increases in ventilation inhomogeneity broadly equivalent to those associated with 15 yr of aging. However, without measurements taken before infection, it is unclear whether the changes result from COVID-19 infection or are risk factors for more severe disease.

## Graphical abstract

**Figure:**
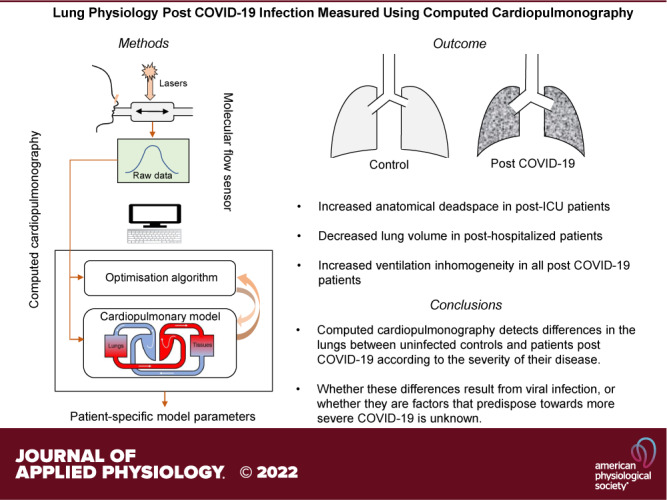

## INTRODUCTION

COVID-19 was declared a pandemic by the World Health Organization on March 11, 2020. By the end of 2021, over 280 million people had contracted COVID-19, resulting in over 5.4 million deaths. In addition, for a significant number of individuals, symptoms may persist for weeks or months after the acute infection has resolved, in a syndrome known as long-COVID. The purpose of the present study was to examine aspects of lung physiology in two cohorts of patients after COVID-19 infection using an approach that we have termed computed cardiopulmonography (CCP).

CCP uses variations in inspired gas tensions to generate dynamic variations in gas exchange. These variations in gas exchange are used within a physiological model of the lung and of the body gas stores to obtain estimates for parameter values that describe aspects of an individual’s respiratory and cardiovascular function. The models used here have been developed around considerations of mass balance and comprise one for gas exchange within an inhomogeneous lung, known as the log-normal lung (1), coupled to a second model for the dissolved (or reversibly reacted) gas stores in the body tissues, known as the circulatory and body gas stores model (2). Both models use a physicochemical model of blood (3) for the circulatory exchange of gases. For CCP to work, highly precise, highly time-resolved measurements of gas exchange are required. Without highly precise measurements, a significant integration error would occur within the modeling that would result in the model lung either collapsing or ballooning to an unphysiological volume over time. This measurement precision has been achieved through the use of laser absorption spectroscopy to provide rapid and highly precise gas analysis across the mainstream respired gas flow (4, 5).

The two post-COVID-19 cohorts we studied were a cohort of military personnel (MCOVID) and a cohort derived from the general patient population [Capturing MultiORgan Effects of COVID-19 Lung Physiology (C-MORE-LP)]. CCP enabled the description of some new features of lung physiology in post-COVID-19 patients that had not been revealed with standard clinical lung function tests. However, in the absence of control measurements made before infection, it is difficult to determine whether these features result directly from COVID-19 infection or whether they are actual risk factors associated with the lung that predispose toward more serious disease.

## METHODS

### Study Participants

The participants were recruited as part of two separate studies. Both studies were conducted in accordance with the general principles of the Declaration of Helsinki, with all participants giving written informed consent.

#### MCOVID cohort.

This study formed part of a wider study in British military personnel (MCOVID) of the effects of COVID-19 infection on their subsequent health (6). All patients apart from two had symptoms that began either in 2020 or in January 2021. For the other two patients, one had a symptom onset date in February 2021 and one in April 2021. No patient had received a vaccination for COVID-19 in advance of the onset of symptoms. The cohort also contained a number of healthy control subjects who had not had symptoms of COVID-19 infection and who had negative antibody tests. For a few individuals who contracted COVID-19 very early in the pandemic before widespread laboratory testing was available, the diagnosis was based on clinical history together with the radiological appearance of the lungs. For all other patients, the diagnosis was confirmed by laboratory testing. The participants were grouped by severity of acute infection into *1*) Control: those who had no evidence of having had COVID-19; *2*) Community: those who had COVID-19 and were managed in the community; *3*) Ward: those who had COVID and were managed in hospital without admission to an intensive care unit (ICU); and *4*) ICU: those who had COVID-19 and were admitted to an ICU for management of the disease. All but two of those admitted to ICU underwent invasive mechanical ventilation (IMV), and one also received extracorporeal membrane oxygenation (ECMO). The study was approved by the Ministry of Defence Research Ethics Committee (reference number: 1061/MODREC/20).

#### C-MORE-LP cohort.

Participants were patients who had been hospitalized with COVID-19 pneumonia and who were recruited from a dedicated post-COVID-19 follow-up outpatient clinic in the Oxford University Hospitals NHS Foundation Trust. The date of admission to hospital with COVID-19 for the patients varied between March 2020 and February 2021. Participants were grouped by severity of acute infection into *1*) Basic/O_2_: patients who had COVID-19 and were managed in hospital with a maximum of simple O_2_ therapy; 2) HFNC/CPAP: patients who had COVID-19 and were managed in hospital with maximum therapy of O_2_ either via a high-flow nasal cannula (HFNC) or via continuous positive airway pressure (CPAP); and 3) IMV: patients who had COVID-19 and were managed with invasive mechanical ventilation (IMV). The study, C-MORE-Lung Physiology, was approved by the North West–Preston Research Ethics Committee (reference number: 20/NW/0235).

### Protocol

#### MCOVID cohort.

Participants were first studied at a median of 22 wk after recovering from COVID-19. A subset of participants were invited to undertake a second study ∼6 mo after their first visit. On each occasion, a set of basic clinical lung function tests (spirometry and gas transfer measurements) were performed. These measurements included the forced expired volume in 1 s (FEV_1_); the forced vital capacity (FVC); the diffusing capacity of the lungs for carbon monoxide corrected for hemoglobin (Dl_CO_c); the carbon monoxide transfer coefficient for the lungs corrected for hemoglobin (KCOc); and the single-breath estimate of alveolar volume at maximum lung capacity. At their first visit patients, but not control participants, underwent a computed tomography (CT) scan of their lungs. Each CT was scored for the presence or absence of ground-glass opacities and the presence or absence of signs of fibrosis.

For the CCP study, each participant breathed through a mouthpiece connected to the measuring device for a period of ∼12 min, during which time their nose was occluded. For the first 7 min the participant breathed air, and for the final 5 min the participant breathed pure O_2_. In most participants, an arterial blood gas sample was drawn during the air-breathing phase of the measurement but not until after at least 90 s of breathing on the mouthpiece had elapsed. In these participants, before the start of the measurement period, the tissue surrounding the radial artery at the wrist was infiltrated with local anesthetic so that the sample could be withdrawn with the minimum of disturbance.

#### C-MORE-LP cohort.

For the C-MORE-LP cohort, patients were first studied at ∼6 mo after discharge from hospital. A subset of patients were invited to undertake a second study ∼1 yr after discharge. Basic clinical lung function tests (spirometry and gas transfer measurements) were performed in association with the first study, though not necessarily on the same day. Most patients also underwent a CT study of their lungs in association with the first visit.

The protocol for the CCP study for the C-MORE-LP cohort was similar to that used with the MCOVID cohort, with participants breathing air for the first 7 min and pure O_2_ for the final 5 min. However, no arterial blood gases were drawn from the C-MORE-LP cohort.

### Molecular Flow Sensor

**Figure 1.**
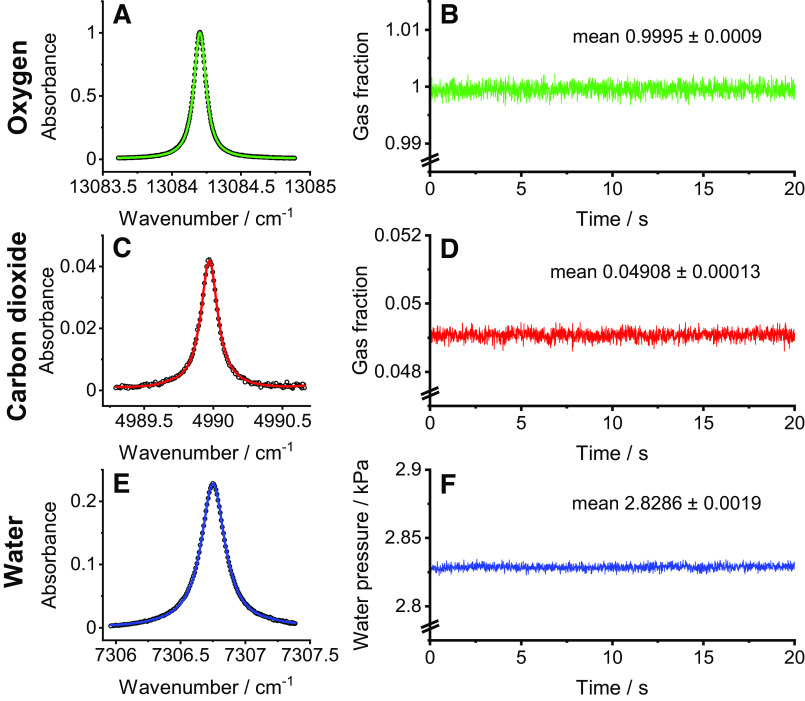
Precision and stability of laser spectroscopy systems for analysis of respired gas every 10 ms. *A, C*, and *E*: typical absorbance spectrum taken within a single 10-ms measurement period for O_2_, CO_2_, and water vapor, respectively. Symbols illustrate data, and curves illustrate the Voigt profiles fitted to the data. *B, D*, and *F*: analyses every 10 ms during 20-s periods of steady gas flows for the O_2_, CO_2_, and water vapor analyzers, respectively. For O_2_ and water vapor, the SD for the measurement is <1/1,000th of the mean. For CO_2_, it is <3/1,000th of the mean.

The measurement system used is called the molecular flow sensor (MFS) and has been described previously (4). It comprises a gas analysis system combined with a purpose-built flow measurement system. The gas analysis system uses laser absorption spectroscopy to measure gas composition within the main respired gas stream. Three near-infrared diode lasers were tuned to scan over absorption features that were specific to O_2_, CO_2_, and water vapor, respectively. Beer’s law was used in combination with a known path length and cross section for the absorption feature of interest to calculate the gas concentrations. For the O_2_ measurement, because the cross section was 2–3 orders of magnitude smaller than for CO_2_ or water vapor, an optical cavity was used to increase the effective path length to gain the required sensitivity. The path length for this was calculated by flowing pure (N5 grade) O_2_ through the cavity to provide a known concentration. Total pressure and gas temperature were measured continuously within the device, and this allowed the combined concentrations of all gas species to be calculated with the ideal gas law. The N_2_ concentration (which was taken to include the Ar in air) could then be calculated by subtraction of the O_2_, CO_2_, and water vapor concentrations from the combined gas concentration. Figure 1 illustrates the precision and stability of the present laser spectroscopy systems used to analyze the O_2_, CO_2_, and water vapor contents of the respired gas every 10 ms.

The flow sensor comprises a purpose-built pneumotachograph constructed from two meshes that enclose the optical paths of the lasers through the gas. As the temperature, pressure, and gas composition are known for the respired gas as it passes through the pneumotachograph meshes, the density and viscosity of the gas can be updated continuously, and this allows a precise calculation of flow.

The overall device was interfaced to a PC through a USB connection, which allowed measurements of flow and gas concentrations to be recorded every 10 ms.

### Model Structure and Parameter Estimation Process

**Figure 2.**
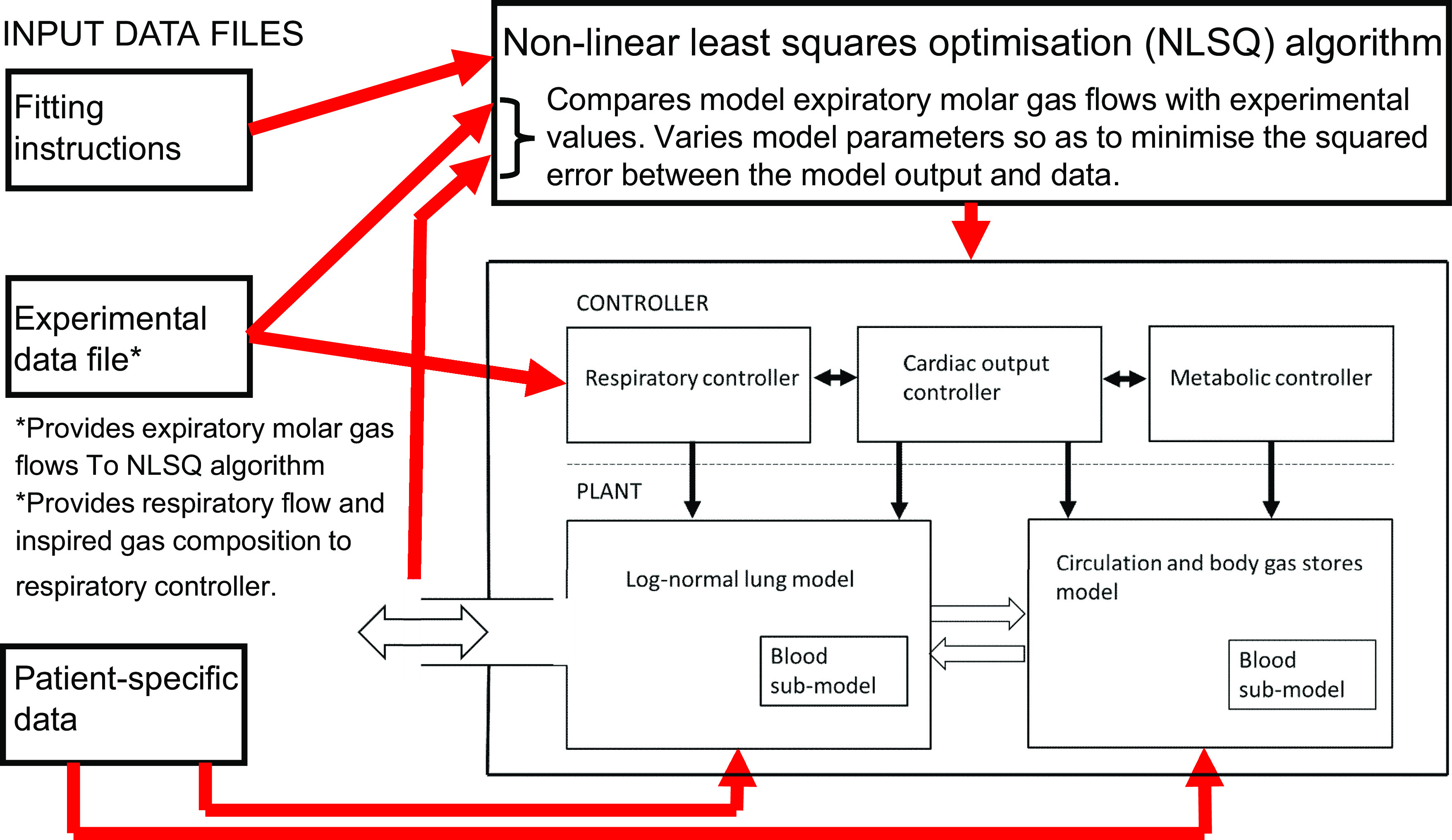
Schematic of model and parameter estimation process. The plant is comprised of 3 core models, 1 for the blood gas dissociation curves, 1 for the inhomogeneity in the lungs, and 1 for the circulation and the gases dissolved in the body tissues. Metabolism drives the model and determines (along with body size) the cardiac output. Total respiratory flow and inspired gas composition are as recorded during the measurement. The model is embedded in a nonlinear least-squares optimization algorithm. This progressively adjusts the parameters of the model so as to minimize the sum of squared deviations between the model and the data for the molar flows for each of the individual gas species during expiration.

To extract physiological meaning from the 10-ms data stream provided by the MFS, a physiological model of lung inhomogeneity was fit to the data. The model employed here was constructed by combining three previously published models, one for the carriage of O_2_ and CO_2_ by the blood (3), one to describe inhomogeneity in the lung which is called the log-normal lung (LNL) model (1), and one to describe the dissolved gas stores which is called the circulation and body gas stores (CBGS) model (2). The model and parameter estimation process are illustrated in Fig. 2. The three models referred to above are shown as the plant in the figure. The metabolic consumption of O_2_ and production of CO_2_ essentially drive most of the model. Metabolic rate is used as the principal determinant of cardiac output (the other being body size). However, total respiratory flow for the model during both inspiration and expiration was derived entirely from the experimentally recorded data, as was the inspired gas composition. The whole model sits within a nonlinear least-squares optimization algorithm. The parameters of the model to be estimated are progressively adjusted by the algorithm so as to minimize the sum of the squared errors for each 10-ms period between the molar flows of the individual gas species expired by the model and those recorded in the experimental data file.

In the study by Mountain et al. (1) that devised the LNL model, there was no CBGS model, and so the gas contents for the pulmonary arterial blood flowing back into the lung were unknown. They therefore had to be estimated as parameters of the model, and in that study there were parameters for the starting mixed venous (pulmonary arterial) concentrations for O_2_ and CO_2_ and for a linear trend to describe how they changed over time. In the present study, there is no need for these parameters because the blood gas contents are determined by the CBGS model that connects the pulmonary venous outflow back through the body gas stores to the pulmonary arterial inflow. The parameters that replace them are the metabolic rate of O_2_ consumption by the tissues (V̇o_2_), the respiratory quotient (R), and the ideal Pco_2_ (Pico_2_), which is used to determine the initial CO_2_ and O_2_ contents within the model at start-up.

One practical problem in studying participants who are essentially naive to gas exchange studies is that wearing a noseclip and breathing through a mouthpiece typically generates a degree of hyperventilation that then progressively alters the body gas stores. Mountain et al. (1) approached this problem by not fitting data from the first 5 min of breathing on the apparatus and fitting trend terms to the mixed venous CO_2_ and O_2_ contents for the period thereafter. In the present study, we were careful to ensure that participants were given time to settle fully into a resting steady state before asking them to breathe on the apparatus. However, we then fitted the model from the first fully recorded breath onward and used the combination of the LNL and CBGS models to track any changes induced through hyperventilation in the body gas stores. This approach enabled a significant reduction in the duration of the test.

Mountain et al. (1) included a measured apparatus dead space volume between the mouth and the measurement plane of the MFS. This principally related to the disposable bacterial filter/heat and moisture exchanger. In the present study, we also included a model of the dispersion (longitudinal mixing) induced within this apparatus dead space volume. The dispersion was determined by computational fluid dynamic simulations that were outsourced to a commercial company (TotalSim, Brackley, UK). A mesh model of the upper airway (courtesy of Prof. J. Wild, University of Sheffield) obtained through MRI imaging was connected at the mouth to the geometric model of the apparatus. The dispersion, which was relatively constant, was then calculated for a range of different steady flow rates that could reasonably be associated with breathing under resting conditions.

Apart from modeling the dispersion introduced by the apparatus dead space, one other refinement was introduced in relation to the anatomical dead space of the lung. In the original study, Mountain et al. (1) used a dead space volume that did not vary with lung volume and employed a normal distribution to describe the inhomogeneity present in the dead space volumes serving the different compartments of the lung. In the present study, we introduced a parameter to allow the anatomical dead space to increase with lung volume, to reflect this well-established feature of the lung. This model parameter, δsVd, could take values that range between 0 (rigid dead space) and 1 (dead space compliance equal to alveolar compliance).

The addition of a further parameter for the dead space compliance introduced the possibility of overparameterizing the model. This was tested for by using the Akaike information criterion (using the number of breaths, rather than the number of 10-ms values, as the number of observations), which balances any improvement in fit against a cost for increased model complexity. On average, the introduction of compliance within the dead space reduced the mean squared error by 4%. This reduced the Akaike information criterion and so was retained within the model.

The model was fit on the ARCUS-HTC system of the University of Oxford’s Advanced Research Computing (ARC) facility. Apart from one or two numerically intensive subroutines that had been written in C++ and precompiled, the models were written in MATLAB. In general, it took between 1 and 2 h on a single multicore processor to obtain a solution.

A list of the parameters estimated within the model is given in Table 1.

**Table 1.** Parameters estimated within the fitting process

VD/ liters (BTPS)	Dead space at functional residual capacity (FRC)
δsVd	Fractional expansion of dead space relative to fractional expansion of alveolar space
σVd	Standard deviation for the standardized dead space
Va/ liters (BTPS)	Alveolar volume at FRC (FRC = Vd + Va)
σlnCl	Standard deviation for the natural logarithm for the standardized lung compliance
σlnCd	Standard deviation for the natural logarithm for the standardized lung vascular conductance (actually estimated as the ratio σlnCd/σlnCl)
V̇o_2_/ liters (STPD)/min	Oxygen consumption
R	Respiratory quotient
Pico_2_/ kPa	Ideal Pco_2_ at initialization of model

### Statistical Analysis

Data from the MCOVID cohort were analyzed separately from those for the C-MORE-LP cohort, but in each case the statistical analysis was undertaken with linear mixed-effects modeling. The participant characteristics included in the model were sex, age, height, body mass index (BMI), and pack years of smoking. Characteristics were excluded if they had already been controlled for within the dependent variable [e.g., age, height, and sex were excluded in the case when the dependent variable was functional residual capacity (FRC) % predicted, because the predicted value is based on these characteristics]. COVID severity was included as a factor in the model. Also included in the model was time [ln(days)] following the onset of symptoms (MCOVID study) or discharge from hospital (C-MORE-LP study), together with the interaction between COVID severity and time. To cater for control participants (MCOVID study) for whom there was no symptom onset or discharge date from hospital, the ln(days) values for the post-COVID participants were adjusted so that the overall mean was zero, and then a value of zero was assigned to all control participants. Participants were included as a random factor. Thus, the linear mixed-effects model for variable *X* could be written as follows:

$$\begin{array}{l} X\;∼\text{ Is female}+\text{age}+\text{height}+\text{ln}\left(\text{BMI}\right)\;+\text{ pack years}+\text{COVIDSeverity}+\text{ln}\left(\text{days}\right)\;+\text{COVIDSeverity }*\text{ ln}\left(\text{days}\right)+\left(\text{1}/\text{participant}\right) \end{array}$$

Nonsignificant factors and covariates relating to participant characteristics and time were removed sequentially, least significant first, until only the significant terms plus COVIDSeverity remained, from which the significance of the COVIDSeverity factors could be judged.

To explore the significance or otherwise of the radiological findings, the above analysis was repeated, but with COVID severity replaced by a radiology score. The radiology score had three levels: *1*) normal CT; *2*) ground-glass opacities present on CT but no evidence of fibrosis; and *3*) one or more features of fibrosis present on CT. For this analysis, we assumed that the radiology of all control participants in the MCOVID study was normal.

The regressors were constructed so that the intercept value was for a male of the mean age, height, and ln(BMI) of the participants across both studies. This was achieved by subtracting the means from each of the individual values for age, height, and ln(BMI). This has no effect on the statistical significance of any term but makes the intercept values more comparable between the MCOVID and C-MORE-LP studies.

## RESULTS

### Participants

#### MCOVID cohort.

At the first study visit, 112 participants undertook the CCP measurement protocol, with 103 producing results that could be included in the analysis. For the second study visit, 67 participants undertook the measurement protocol, with 65 producing results that could be used. Reasons for being unable to use the results in the analysis included syncope induced by the arterial blood gas sampling (2), gas leakage around the mouthpiece and/or noseclip (4), together with a few data sets (5) to which the model could not fit for reasons that were unclear. A summary of the participants’ physical characteristics by disease severity is given in Table 2 for both the initial and the follow-up study. This table also provides the range of COVID-19 severity scores associated with each group, as defined through a World Health Organization scale (7). Participants were mostly male, they were well matched across disease severity for age and height, but weight and BMI were higher in those who had had more severe disease.

**Table 2.** MCOVID: participant characteristics

	Control	Community	Ward	ICU	Overall
WHO severity	0	1–3	4–6	7–9	0–9
Characteristics					
No. of participants					
1st visit	22	52	30	8	112
2nd visit	10	34	17	6	67
Female/ %					
1st visit	3 (14)	7 (13)	4 (13)	0 (0)	14 (13)
2nd visit	3 (30)	4 (12)	2 (12)	0 (0)	9 (13)
Age/ yr					
1st visit	39 ± 9	37 ± 9	42 ± 8	44 ± 13	39 ± 9
2nd visit	41 ± 7	39 ± 10	40 ± 9	47 ± 8	40 ± 9
Height/ m					
1st visit	1.77 ± 0.07	1.79 ± 0.10	1.76 ± 0.06	1.78 ± 0.08	1.78 ± 0.08
2nd visit	1.76 ± 0.09	1.80 ± 0.10	1.77 ± 0.06	1.76 ± 0.06	1.78 ± 0.09
Weight/ kg					
1st visit	79 ± 9	91 ± 17***	96 ± 13****	100 ± 18***	90 ± 16
2nd visit	80 ± 10	92 ± 17	96 ± 15	99 ± 15	92 ± 16
BMI/ kg·m^−2^					
1st visit	25 ± 2	28 ± 3***	31 ± 4****	32 ± 5****	29 ± 4
2nd visit	27 ± 4	29 ± 3	31 ± 4	32 ± 5	29 ± 4
Smoking/ %					
1st visit	3 (14)	5 (10)	9 (30)	6 (75)	23 (20)
2nd visit	1 (10)	5 (15)	6 (35)	4 (67)	16 (24)

Values are means ± SD. BMI, body mass index; ICU, intensive care unit; WHO, World Health Organization. ***P < 0.005, ****P < 0.001.

#### C-MORE-LP cohort.

Sixty-six participants undertook the CCP measurement protocol, with 56 producing results that could be included in the analysis. Of the failures, six participants were unable to complete the breathing test and in four cases the model failed to fit the data. Of the 66 participants, 30 returned for a second visit. For this visit the model fitted 28 of the data sets successfully and there were 2 failures. A summary of the participants’ characteristics by disease severity is given in Table 3 for both the initial and the follow-up study. This study did not include control participants. The patients were well matched across disease severity for age and height, but weight and BMI increased with disease severity.

**Table 3.** C-MORE-LP: participant characteristics

	Basic/O_2_	HFNC/CPAP	IMV	Overall
WHO severity	4–5	6	7–9	4–9
Characteristics				
No. of participants				
1st visit	26	26	14	66
2nd visit	11	16	3	30
Female/ %				
1st visit	8 (31)	4 (15)	4 (29)	16 (24)
2nd visit	1 (9)	1 (6)	2 (67)	4 (13)
Age/ yr				
1st visit	60 ± 9	59 ± 9	61 ± 9	60 ± 9
2nd visit	59 ± 6	59 ± 11	60 ± 9	59 ± 9
Height/ m				
1st visit	1.74 ± 0.11	1.73 ± 0.08	1.69 ± 0.11	1.72 ± 0.10
2nd visit	1.80 ± 0.09	1.75 ± 0.07	1.55 ± 0.07	1.75 ± 0.10
Weight/ kg				
1st visit	87 ± 17	97 ± 23	97 ± 28	93 ± 22
2nd visit	100 ± 15	96 ± 23	95 ± 40	98 ± 22
BMI/ kg·m^−2^				
1st visit	28 ± 5	32 ± 7	33 ± 9	31 ± 7
2nd visit	31 ± 5	31 ± 7	39 ± 14	32 ± 7
Smoking/ %				
1st visit	9 (35)	11 (42)	7 (50)	27 (39)
2nd visit	5 (45)	6 (38)	1 (33)	12 (40)

Values are means ± SD. Basic/O_2_, standard hospital care with or without simple oxygen therapy; BMI, body mass index; HFNC/CPAP, high-flow nasal cannula or continuous positive airway pressure; IMV, invasive mechanical ventilation; WHO, World Health Organization.

### Clinical Lung Function Parameters

#### MCOVID cohort.

The clinical lung function parameters are given in Table 4. There was little by way of difference between most COVID severity groups for either FEV_1_ % predicted or FEV_1_/FVC. However, FVC % predicted was significantly lower in the ICU group compared with the control participants, and FEV_1_/FVC was significantly higher. No significant changes were detected over time, or over time by severity. Grouping individuals by radiology score rather than COVID severity removed any significant effect.

**Table 4.** MCOVID: spirometry, gas transfer, and radiology

	Control	Community	Ward	ICU	Overall
WHO severity	0	1–3	4–6	7–9	0–9
Variable					
Days from onset of symptoms					
1st visit	N/A	182 ± 94	153 ± 58	160 ± 77	170 ± 82
2nd visit	N/A	372 ± 79	357 ± 62	371 ± 78	367 ± 71
FEV_1_/ % predicted					
1st visit	101 ± 14	94 ± 11*	98 ± 13	90 ± 18	96 ± 13
2nd visit	104 ± 15	95 ± 11	100 ± 13	93 ± 17	97 ± 13
FVC/ % predicted					
1st visit	108 ± 13	102 ± 12	101 ± 12	89 ± 17***	102 ± 13
2nd visit	112 ± 11	103 ± 12	103 ± 12	93 ± 16	103 ± 13
FEV_1_/FVC					
1st visit	0.75 ± 0.05	0.75 ± 0.07	0.78 ± 0.04	0.80 ± 0.03*	0.76 ± 0.06
2nd visit	0.76 ± 0.08	0.75 ± 0.07	0.78 ± 0.04	0.80 ± 0.04	0.77 ± 0.06
Dl_CO_c/ % predicted					
1st visit	97 ± 8	91 ± 18*	84 ± 15***	74 ± 15****	89 ± 16
2nd visit	99 ± 13	87 ± 12	88 ± 12	79 ± 14	88 ± 13
KCOc/ % predicted					
1st visit	99 ± 9	99 ± 12	104 ± 20*	98 ± 9	100 ± 14
2nd visit	99 ± 8	95 ± 10	105 ± 19	101 ± 6	99 ± 13
Alveolar volume/ % predicted					
1st visit	98 ± 10	91 ± 13**	82 ± 17****	75 ± 16****	89 ± 15
2nd visit	99 ± 12	91 ± 11	84 ± 12	77 ± 13	89 ± 13
Ground-glass changes on CT/ %				
1st visit	N/A	0 (0)	5 (19)	3 (43)	8 (11)
Fibrotic changes on CT/ %					
1st visit	N/A	1 (2)	6 (23)	3 (43)	10 (13)

Values are means ± SD. CT, computed tomography; Dl_CO_c, diffusing capacity of the lung for carbon monoxide corrected for hemoglobin; FEV_1_, forced expiratory volume in 1 s; FVC, forced vital capacity; ICU, intensive care unit; KCOc, carbon monoxide transfer coefficient for the lung corrected for hemoglobin; N/A, not available; WHO, World Health Organization. *P < 0.05, **P < 0.01, ***P < 0.005, ****P < 0.001.

Values for Dl_CO_c % predicted and alveolar volume % predicted decreased in a highly significant manner as COVID severity increased. For neither variable was the effect of ln(BMI) significant. For KCOc % predicted, there was no significant relationship with COVID severity. No significant effects were detected for any of the three variables over time or over severity by time. Grouping individuals by radiology score rather than COVID severity removed all significance.

#### C-MORE-LP cohort.

The clinical lung function parameters for this cohort are given in Table 5. No significant differences were detected between severity groups for FEV_1_ % predicted and FVC % predicted, although FEV_1_/FVC was significantly higher for the IMV group compared with control participants. Similarly, no significant differences were present between severity groups for Dl_CO_c % predicted, KCOc % predicted, and alveolar volume % predicted. Grouping individuals by radiology score at the first visit rather than COVID severity revealed a significant decline in alveolar volume % predicted (*P* < 0.05) associated with the presence of fibrosis.

**Table 5.** C-MORE-LP: spirometry, gas transfer, and radiology

	Basic/O_2_	HFNC/CPAP	IMV	Overall
WHO severity	4–5	6	7–9	4–9
Variable				
Days since hospital discharge	154 ± 66	164 ± 50	157 ± 37	158 ± 54
FEV_1_/ % predicted	94 ± 26	92 ± 18	95 ± 18	94 ± 20
FVC/ % predicted	95 ± 22	94 ± 20	90 ± 17	93 ± 20
FEV_1_/FVC	0.76 ± 0.08	0.76 ± 0.08	0.81 ± 0.04*	0.78 ± 0.07
Dl_CO_c/ % predicted	79 ± 14	80 ± 12	72 ± 19	77 ± 15
KCO_C_/ % predicted	94 ± 18	100 ± 19	95 ± 22	96 ± 19
Alveolar volume/ % predicted	87 ± 15	84 ± 19	77 ± 8	83 ± 15
Ground-glass changes on CT/ %	11 (42)	13 (50)	14 (100)	38 (58)
Fibrotic changes on CT/ %	1 (4)	4 (15)	10 (71)	15 (23)

Values are means ± SD. Basic/O_2_, standard hospital care with or without simple oxygen therapy; CT, computed tomography; Dl_CO_c, diffusing capacity of the lung for carbon monoxide corrected for hemoglobin; FEV_1_, forced expiratory volume in 1 s; FVC, forced vital capacity; HFNC/CPAP, high-flow nasal cannula or continuous positive airway pressure; IMV, invasive mechanical ventilation; KCOc, carbon monoxide transfer coefficient for the lung corrected for hemoglobin; WHO, World Health Organization. *P < 0.05.

### Fit of LNL-CBGS Model

**Figure 3.**
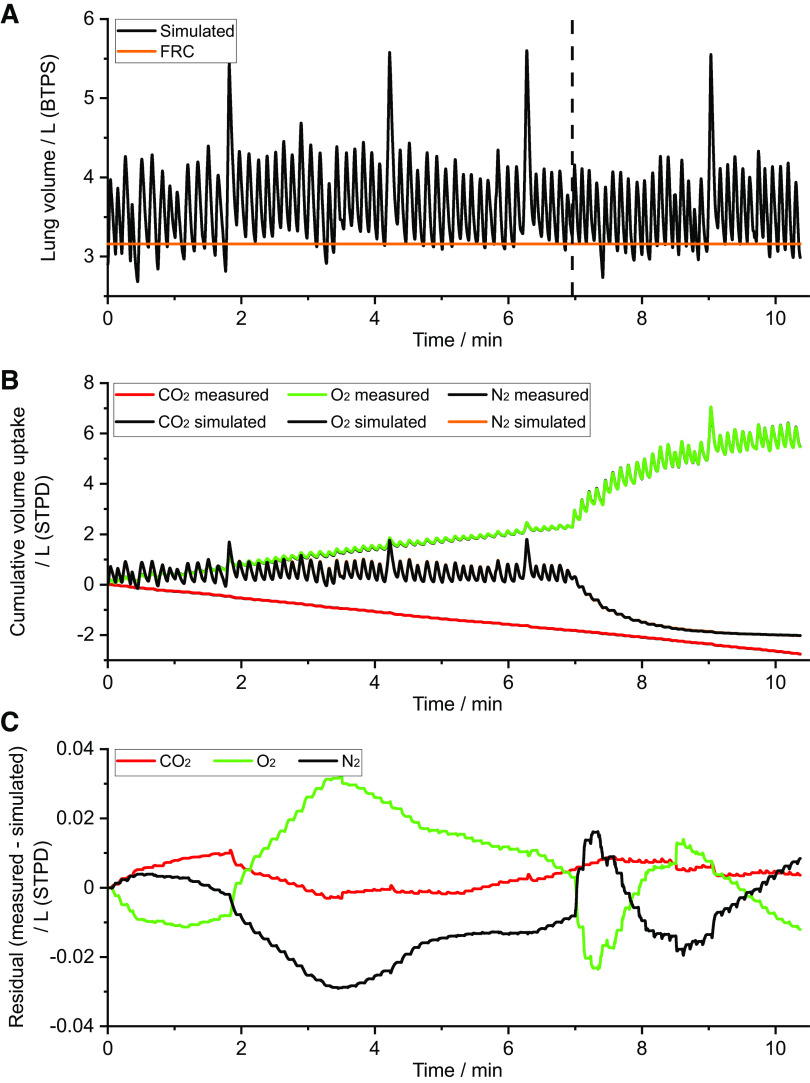
Example fit of model to gas exchange data. *A*: calculated lung volume against time. FRC, functional residual capacity. *B*: measured and model-simulated flows of individual gas species at the mouth. The model responses are essentially hidden as they are overlaid by the data. *C*: cumulative residuals (measured minus simulated) illustrating the residual error in the model fit for the gas exchange data.

**Figure 4.**
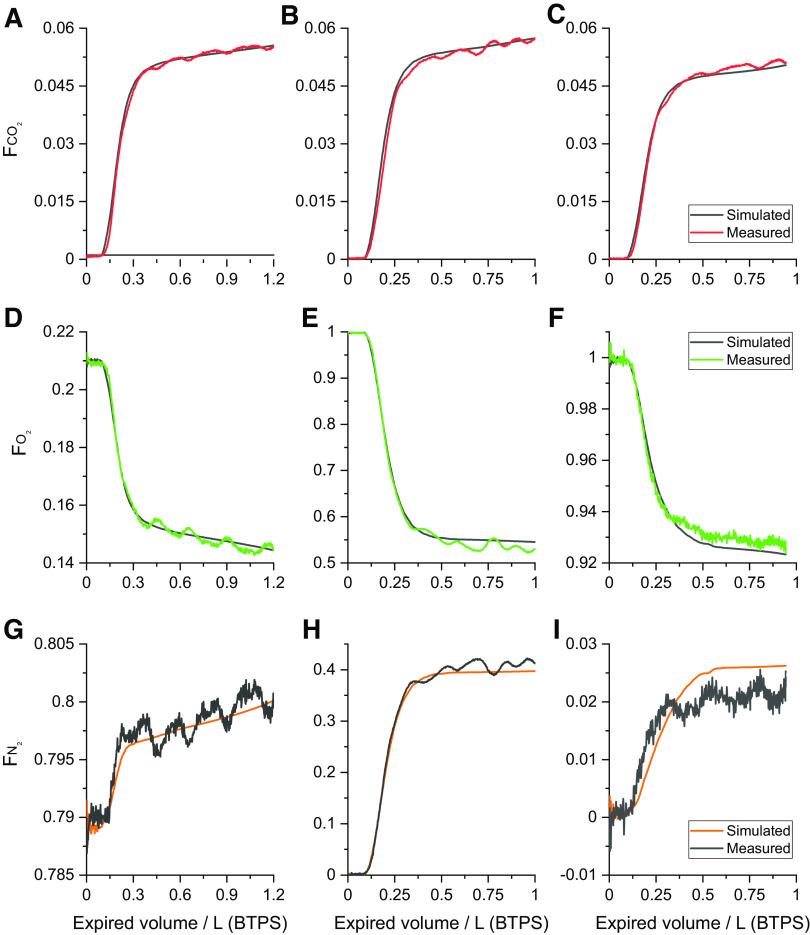
Expirograms for CO_2_, O_2_, and N_2_ fractions (F) for a breath during the air-breathing phase and for a breath early and late in the N_2_ washout phase, for an example data set. *A–C*: CO_2_ expirograms. *D–F*: O_2_ expirograms. *G–I*: N_2_ expirograms. *A*, *D*, and *G*: breath during air-breathing phase. *B*, *E*, *H*: breath during early N_2_ washout phase. *C*, *F*, and *I*: breath during late N_2_ washout phase.

An example of the fit of the model to the data is shown in Fig. 3. The calculated lung volumes show some variation in FRC but no overall trend toward an increasing or decreasing volume over time. For the tidal flow of the individual gas species at the mouth, the model predictions and recorded data are essentially indistinguishable from one another when plotted on the same axes. Some discrepancies between model and data can be observed in the plots of cumulative residuals illustrating that the model does not fit perfectly, but quantitatively these are very small. Figure 4 illustrates example expirograms from a breath during the air-breathing phase, from a breath early in the N_2_ washout phase, and from a breath late in the N_2_ washout phase. One distinctive feature is the cardiogenic oscillations recorded in the data that are not present in the model. The model can be seen to track the shape of the expirogram very well, although there is a small degree of misfit in the plateau phase for O_2_ and N_2_ late in the N_2_ washout phase.

**Figure 5.**
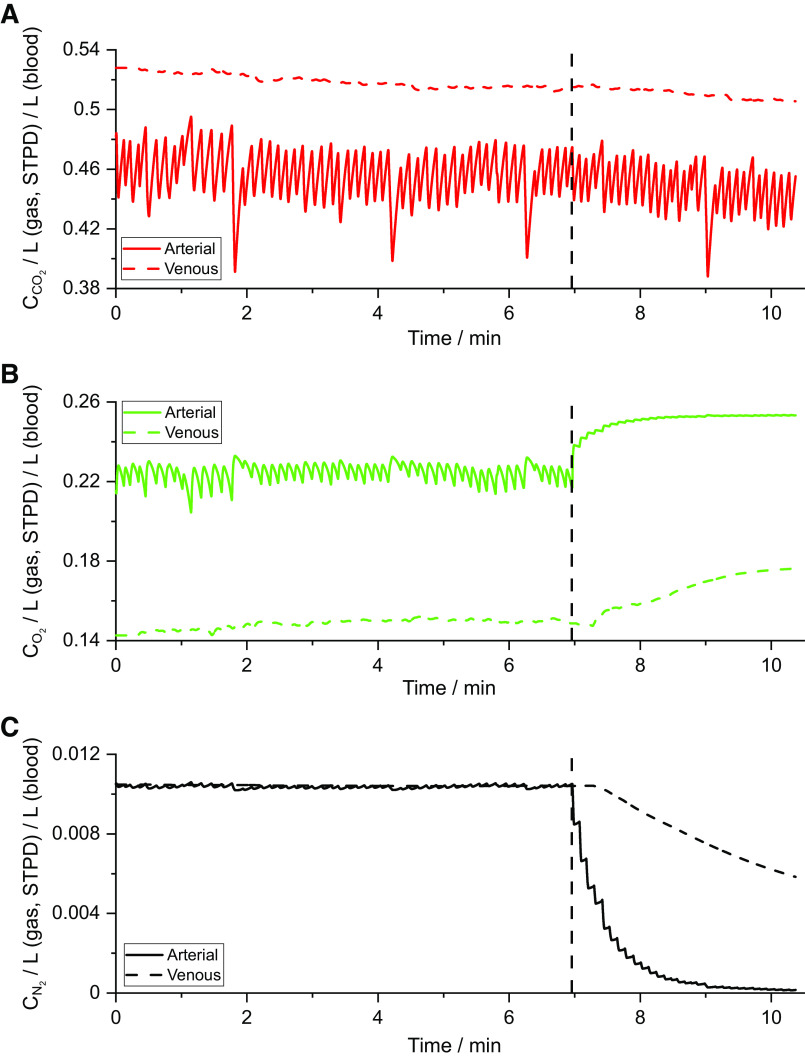
Calculated blood gas contents (C) derived within the model for an example data set. *A*: systemic mixed venous and arterial contents for CO_2_. *B*: systemic mixed venous and arterial contents for O_2_. *C*: systemic mixed venous and arterial contents for N_2_.

Once fitted, the model provided calculated values for the partial pressures and contents for CO_2_ and O_2_ in the blood throughout the test, and an example record for the gas contents is shown in Fig. 5. This volunteer exhibited a mild degree of hyperventilation as evidenced by the progressive decrease in the mixed venous CO_2_ content. The variations toward and away from asphyxia with breathing are clearly illustrated, as are the effects of breathing pure O_2_. For the MCOVID cohort, arterial blood gas samples were drawn during the air-breathing phase of the protocol from most participants. The degree to which these coincide with the contemporaneous values calculated by the model provides a useful validation for how well the model is performing. With an ∼30-s window centered on a time 10 s earlier than the arterial sampling time point (each window was adjusted slightly in length so as to cover an integral number of breathing cycles) the overall difference in Pco_2_ between data and model was −0.01 ± 0.38 (mean ± SD) kPa and the overall difference in Po_2_ between model and data was −0.60 ± 1.44 kPa.

### Model Parameters

#### MCOVID cohort.

**Figure 6.**
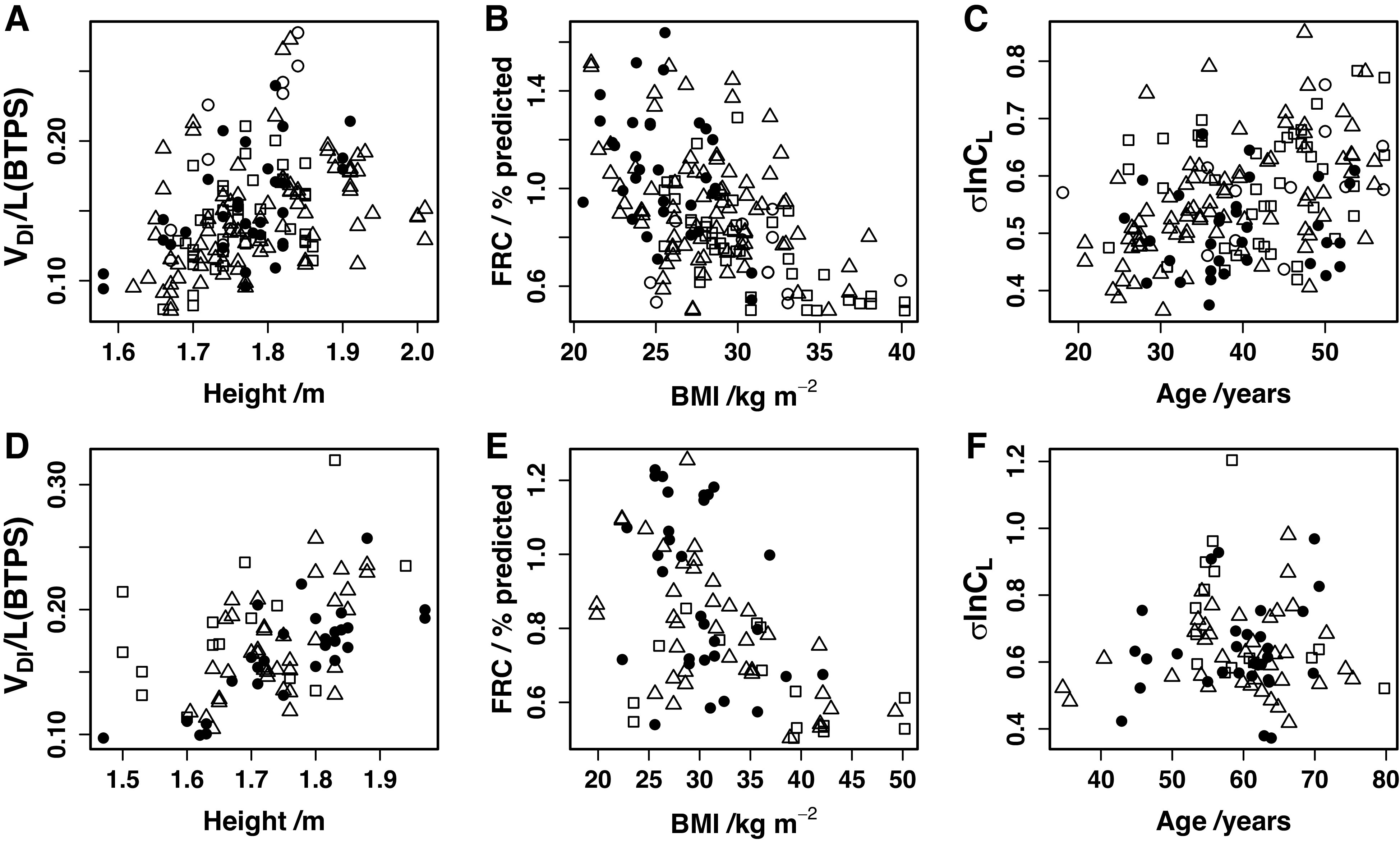
Influence of selected physical characteristics of participants on selected parameter estimates. *A–C*: MCOVID cohort (112 participants). *D–F*: C-MORE-LP cohort (66 participants). *A* and *D*: influence of height on end-inspiratory anatomical dead space (Vdi) in liters (BTPS). *B* and *E*: influence of body mass index (BMI) on functional residual capacity (FRC) % predicted. *C* and *F*: influence of age on the SD for the natural logarithm of the standardized lung compliance (σlnCl). MCOVID cohort: ●, Control group; △, Community group; □, Ward group; ○, ICU group. C-MORE-LP cohort: ●, Basic/O_2_ group; △, HFNC/CPAP group; □, IMV group. After controlling for other factors, the level of statistical significance for each significant relationship illustrated is given in Table 6.

Details for the linear mixed-effects modeling are shown in Table 6 for the selected parameters of anatomical dead space at the end of a standard inspiration (Vdi; essentially equivalent to the Fowler dead space); functional residual capacity (FRC), % predicted; and the standard deviation for the log of the standardized lung compliance, σlnCl. This table illustrates the influence that certain physical characteristics had on the parameter values, and Fig. 6 shows this for the effect of height on Vdi, BMI on FRC % predicted, and age on σlnCl. A summary of the fitted values for all the model parameters for this cohort is given in Table A1. Vdi was significantly larger for the ICU group compared with the Control group. Values for FRC % predicted were smaller in the Ward and ICU groups compared with the Control group. Values for σlnCl were significantly greater in the Community and Ward groups compared with the Control group. There were no significant effects of time since onset of symptoms for these variables. Grouping individuals by radiology rather than by COVID severity revealed no significant association with any variable. Although not identical, the results for the model alveolar volume at FRC (Va) were qualitatively similar to those for FRC % predicted (i.e., Va decreased with increasing severity of illness), and, although not reaching statistical significance, the results for (end-expiratory) anatomical dead space (Vd) appeared qualitatively similar to those for Vdi.

**Table 6.** Regression coefficients for COVID-19 severity and for significant participant characteristics for selected variables from the linear mixed-effects modeling

MCOVID Cohort					C-MORE-LP Cohort				
Predictors	Est.	SE	df	*P*	Predictors	Est.	SE	df	*P*
*Vdi, liter*									
Intercept	0.160	0.0073	110	**<0.001**	Intercept (4–5)	0.165	0.007	62	**<0.001**
Severity: 1–3	−0.004	0.0077	106	0.640	Severity: 6	0.010	0.009	69	0.274
Severity: 4–6	−0.002	0.0092	105	0.824	Severity: 7–9	0.040	0.012	59	**0.002**
Severity: 7–9	0.028	0.0130	100	**0.031**	Height/ m	0.262	0.044	53	**<0.001**
Is female	−0.026	0.0090	128	**0.004**					
Age/ yr	0.002	0.0003	105	**<0.001**					
Height/ m	0.152	0.0389	105	**<0.001**					
ln(BMI)	−0.060	0.0225	103	**0.009**					
*FRC, % predicted*									
Intercept	95.8	4.9	107	**<0.001**	Intercept (4–5)	85.4	3.5	65	**<0.001**
Severity: 1–3	−6.4	5.4	106	0.235	Severity: 6	−5.5	4.3	77	0.207
Severity: 4–6	−15.8	6.5	106	**0.018**	Severity: 7–9	−14.9	6.1	59	**0.018**
Severity: 7–9	−21.6	9.3	102	**0.022**	ln(BMI)	−45.0	11.1	60	**<0.001**
ln(BMI)	−74.8	15.8	105	**<0.001**	Pack years	0.4	0.2	54	**0.016**
*σlnCl*									
Intercept	0.530	0.018	107	**<0.001**	Intercept (4–5)	0.61	0.03	63	**<0.001**
Severity: 1–3	0.054	0.020	108	**0.008**	Severity: 6	0.01	0.04	72	0.870
Severity: 4–6	0.065	0.022	107	**0.005**	Severity: 7–9	0.08	0.05	57	0.101
Severity: 7–9	0.026	0.036	98	0.473	Is female	0.10	0.05	54	**0.037**
Age/ yr	0.004	0.001	101	**<0.001**					
Pack years	0.005	0.002	90	**0.012**					

df, degrees of freedom; Est, estimate; FRC, functional residual capacity; *P*, probability value; Vdi, anatomical dead space at end of a standard inspiration (analogous with the Fowler dead space); σlnCl, standard deviation for the natural logarithm of the standardized lung compliance. Significant *P* values are in bold.

#### C-MORE-LP cohort.

Details for the linear mixed-effects modeling are shown in Table 6 for the selected parameters of Vdi, FRC % predicted, and σlnCl. The analysis demonstrated the influence of certain physical characteristics on the parameter values, and Fig. 6 illustrates this for the effect of height on Vdi, BMI on FRC % predicted, and age (not significant) on σlnCl. A summary of all the fitted model parameters for this cohort is given in Table A2. Vdi was significantly larger for the IMV group compared with the Basic/O_2_ group. FRC % predicted was significantly lower for the IMV group compared with the Basic/O_2_ group. Although not reaching statistical significance, the findings for Va appeared similar to those for FRC % predicted. σlnCl was not significantly altered across severity groups. There were no significant effects of time since hospital discharge on these variables. Grouping individuals by radiology rather than by COVID severity revealed no significant association with any variable apart from Vdi, where the presence of fibrosis predicted a greater value for Vdi. Comparing the two fits, however, the Akaike information criterion was lower when individuals were grouped by COVID severity rather than by radiology, indicating that COVID severity was a better predictor for Vdi than radiology.

### Blood Gas Data: MCOVID Cohort

The arterial blood gas results for the MCOVID cohort are given in Table A3, along with a set of variables calculated by the model. Arterial Po_2_ ($\text{P}{\text{a}}_{{\text{O}}_{2}}$) was significantly lower for the ICU group compared with the Control group, and the ideal alveolar to arterial (iA–a)Po_2_ gradient was significantly greater. However, these were not large effects. There were no significant effects for any other variable, and there were no significant effects over time. There were also no significant effects when participants were grouped by radiology score instead of by COVID severity.

### Comparison between MCOVID and C-MORE-LP Cohorts

**Figure 7.**
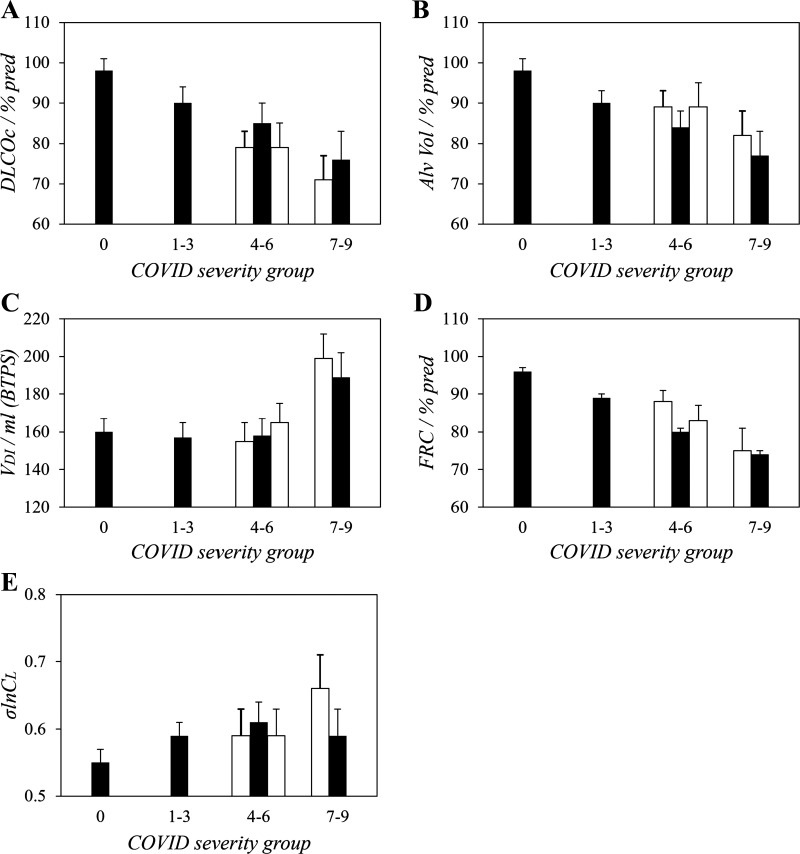
Comparison between the MCOVID and C-MORE-LP cohorts of the values for selected parameters at the different severity levels for prior COVID-19 infection. *A*: lung diffusing capacity for CO corrected for hemoglobin (Dl_CO_c). *B*: alveolar volume (Alv Vol). *C*: end-inspiratory anatomical dead space (Vdi). *D*: functional residual capacity (FRC). *E*: standard deviation for the natural logarithm of standardized lung compliance (σlnCl). Values have been corrected to those for a standard participant who is male, nonsmoking and with an age of 47.5 yr, height of 1.76 m, and body mass index (BMI) of 29.5 kg·m^−2^. Filled bars, MCOVID cohort; open bars, C-MORE-LP cohort. Error bars are 1 SE. % pred, % predicted.

No formal statistical comparison between the results from the two cohorts has been attempted. However, Fig. 7 illustrates, for selected variables, values for each severity group predicted for a male, nonsmoking, but otherwise average participant (47.5 yr, 1.76 m, and BMI of 29.5 kg·m^−2^) based on the findings from the linear mixed-effects modeling for each cohort. The figure illustrates the much narrower range of severity groups available for comparison within the C-MORE-LP cohort compared with those available within the MCOVID cohort. Within this, however, both the absolute values within severity groups and the direction of change across severity groups appear very similar across the two cohorts.

## DISCUSSION

This study applied a new technological approach that has been named computed cardiopulmonography (CCP) to investigate lung physiology in two cohorts of post-COVID-19 patients. One cohort, MCOVID, was constructed from patients of working age. Apart from patients whose infection had been managed in hospital, this cohort also included both control participants and patients whose infection had been managed in the community. It therefore covered a wide range of severities for the prior acute infection. Many of these patients also provided an arterial blood gas sample while breathing through the MFS during the CCP study protocol. The second cohort, C-MORE-LP, was older and more representative of the general population. It did not include either a control group or patients managed in the community, and there were no blood gas samples associated with it.

### Pathophysiological Findings

For the groups that had been most severely affected by COVID-19 (ICU group for MCOVID, IMV group for C-MORE-LP), CCP revealed an enlarged anatomical dead space. The mean arterial Po_2_ for the ICU group was significantly lower than for the control participants, and the (iA–a)Po_2_ gradient was significantly higher. The radiology for these particular patients commonly revealed a degree of fibrosis. An interesting, but unanswered question is whether these changes result directly from COVID-19 pneumonia or whether they arise as a result of invasive mechanical ventilation.

For those patients who did not require ICU admission or IMV, there was no significant increase in anatomical dead space or any significant decrease in arterial Po_2_ or increase in (iA–a)Po_2_ gradient. These features suggest that there is little by way of abnormality in relation to the efficiency of pulmonary gas exchange. Indeed, this is in keeping with a study of tissue from patients who had previously had COVID-19 and who were undergoing a partial lung resection for unrelated pathologies. In these patients, the histology of the parenchyma surrounding the lesion was found to be normal (8).

In apparent contrast to this conclusion, there has developed a large literature, e.g., Refs. 9–14, surrounding clinical lung function measurements to suggest that significant reductions in Dl_CO_ persist in patients after COVID-19 pneumonia and that this may relate to parenchymal lung disease. In both of our cohorts we also found a reduction in the single-breath Dl_CO_. Furthermore, two recent studies that employed hyperpolarized Xe magnetic resonance imaging to study the lung after COVID-19 found a reduction in the ratio of Xe dissolved inside red blood cells relative to that dissolved in the tissue/plasma (15, 16). In these studies, the imaging sequence was designed so that the signal was very heavily weighted toward red blood cells and tissue that were in close apposition to the alveoli, and so the results suggest some parenchymal abnormality. The second of these studies (16) found a close correlation between the reduction in this ratio and the reduction in Dl_CO_. As acute COVID-19 pneumonia is associated with pulmonary vascular endothelialitis and microthrombi (17), one possibility is that the reductions in Dl_CO_ and in red blood cell-to-tissue Xe ratio were caused through some loss of small vessels and capillaries in the lung. Such changes, however, would not necessarily result in any abnormality under resting conditions in the efficiency of pulmonary gas exchange or in the arterial blood gas values.

Dl_CO_ itself is the product of two primary measures. The first is KCO, which is the rate at which CO disappears from the lung under the standardized condition of a breath hold at total lung capacity (TLC). In essence, this may be seen as a “density” for the alveolar surface area at that volume. The second primary measure is the alveolar volume, which is a single-breath estimate (usually by He dilution) of TLC (this typically will underestimate true TLC by 5–10%). In our study, and in a number of others (see Refs. 9–14), it is this alveolar volume rather than KCO that is low after COVID-19. The interpretation of this is not straightforward. Although we did not detect a significant effect of BMI on this volume from the Dl_CO_ measurements, others have detected some reduction in total lung capacity with increasing BMI (18). If some part of the reduction in alveolar volume were related to obesity, then a “normal” value for KCO should be seen as inappropriate, as the Global Lung Initiative reference value project found that the predictions for Dl_CO_ were essentially unaffected by whether or not data for obese individuals were included (19). Thus for obese individuals with normal lungs, any decrease in alveolar volume below normal would need to be compensated for by an increase in KCO to a supranormal value.

Estimates for FRC were obtained as part of the CCP methodology. These values were divided by predicted values based on the individuals’ age, height, and sex to produce values expressed as % predicted. However, the predictive equations do not include a term for BMI, and as FRC is known to vary substantially with BMI (18), we included the log of the BMI as a covariate in the linear mixed-effects model. Although this term was significant for both cohorts, it did not eliminate a significant association between COVID severity and the reduction in FRC. Figure 7 illustrates the reductions in FRC with severity for a hypothetical participant at a standardized BMI of 29.5 kg·m^−2^, and these were strikingly similar to the reductions observed in alveolar volume from the Dl_CO_ measurements.

There are a number of possible causes for the low FRC and for the low alveolar volume from the single-breath Dl_CO_ washout. The first is that some fraction of the alveolar volume is obstructed or unavailable to inspired gas and is therefore not detected by washout methodologies. However, if there were any pulmonary blood flow associated with such a volume, then this would form a shunt blood flow (very low ventilation-to-perfusion ratio), which would not be consistent with the normal arterial blood gas values that were observed for most participants. A second possibility is that hospital admission for COVID-19 pneumonia is associated with a loss of lung parenchyma. A third possibility is that small lungs are an additional risk factor for developing severe COVID-19 pneumonia. These last two possibilities really cannot be distinguished, and this illustrates very clearly the difficulties in interpretation that arise when there is an absence of measurements before infection to act as the control values.

Ventilation inhomogeneity can be viewed as the unevenness with which the lung inflates and deflates during inspiration and expiration, and our parameter, σlnCl, provides a standard deviation for this property across the volume of the lung. In the MCOVID cohort, the values for σlnCl were higher for the patients than for the control participants. This reached statistical significance for both the Community and Ward groups, although not for the ICU group, where the number of patients studied was considerably fewer. For the C-MORE-LP cohort, there were no significant differences between severity groups, but this may simply reflect the absence of a control group that had no prior infection with COVID-19. Indeed, once the values from both cohorts had been corrected for the effects of sex, age, height, and BMI to reflect values for the standard participant (Fig. 7), the mean values for the non-IMV patients in the C-MORE-LP cohort were extremely similar to those for the MCOVID patients, and some ∼0.05 above the value for the MCOVID control group. This is not a large effect when compared with effect sizes associated with chronic airways disease (1, 20). Nevertheless, in the MCOVID cohort σlnCl increases significantly with age by 0.004/yr (Table 6; also see Fig. 6*C*), and in relation to this specific measure, infection with COVID-19 could be considered to have aged the lung by ∼15 yr.

### Computed Cardiopulmonography

This study is the first use of the full CCP methodology (which includes the model of the circulation and body gas stores) in patients. Apart from FRC, there are no equations to provide predicted values for the model parameters based on a person’s physical characteristics. To control for such variations, we initially included a factor for sex, linear terms for age and height, and a log linear term for BMI in the mixed-effects models. We then subsequently removed them if they were not significant. This exercise demonstrated that a number of these characteristics were important, in particular height for dead space, BMI for FRC % predicted, and age for σlnCl (Fig. 6).

A particular difficulty with conducting respiratory measurements is that the mouthpiece and noseclip used commonly cause a degree of hyperventilation. The model of the circulation and body gas stores contained within CCP compensates for this, which in turn enables the respiratory data to be used for fitting from the very beginning of the test (although the participant should spend some time sitting quietly before starting the test to ensure that the body’s gas stores are reasonably close to a steady state). The effects of hyperventilation can be seen in Tables A1 and A2 from the higher values for the directly measured respiratory exchange ratio (RER) for air breathing compared with the respiratory quotient (R) estimated for the tissues from the model. The difference arises from the extra CO_2_ eluting from the body’s gas stores.

CCP was developed as a noninvasive technology, and a penalty associated with this is the absence of blood-side measurements relating to gas exchange. In relation to this, Mountain et al. (1) noted for the lung that, although the identification of airways parameters (Vd, Va, σlnCl, σVd; see Table 1 for definitions) was straightforward, some of the blood-side parameters were much harder to identify. Indeed, Mountain et al. adopted the approach of estimating the blood gas concentrations for the pulmonary arterial inflow and the standard deviation for the log distribution for standardized pulmonary vascular conductance, σlnCd, and then estimating a cardiac output based on the oxygen consumption and assuming a value from the literature for the correlation for the bivariate log-normal distribution of compliance and vascular conductance in the lung. We have followed this approach in the present study, except that the estimates for the blood gas concentrations flowing into the lung have been replaced by the estimates for V̇o_2_, R, and Pico_2_. The considerable number (126) of blood gas measurements made during the MCOVID protocol has now provided an opportunity to validate how well this approach to the blood-side parameters worked. From Table A3, it can be seen that mean error between the data and the model estimate for arterial Pco_2_ ($\text{P}{\text{a}}_{\text{C}{\text{O}}_{2}}$) is negligible (<0.1 kPa), showing that the estimate is unbiased, but this difference is somewhat larger for $\text{P}{\text{a}}_{{\text{O}}_{2}}$ (∼0.5 kPa). Similarly, the SD for the difference between measurement and model was ∼0.4 kPa for $\text{P}{\text{a}}_{\text{C}{\text{O}}_{2}}$ but was much higher for $\text{P}{\text{a}}_{{\text{O}}_{2}}$ at ∼1.6 kPa. The arterial blood gas samples were not drawn slowly (and therefore did not provide a proper average over a number of respiratory cycles) and there is also some uncertainty as to how best to time align the sample with the model data, and both of these factors will contribute to the SD. Nevertheless, the large SD for the $\text{P}{\text{a}}_{{\text{O}}_{2}}$ difference suggests that the uncertainties in the estimates for the blood-side parameters has degraded somewhat the quality of the model’s prediction for the arterial blood gases.

In conclusion, a novel technique, CCP, has been applied to two cohorts of patients who underwent follow-up after COVID-19 infection. The technique revealed an elevated anatomical dead space in patients who had been admitted to the ICU and/or had undergone IMV compared with a control group of participants who had either milder or no prior infection with COVID-19. The technique indicated that FRC was lower in patients who had been hospitalized with COVID-19 or who had had more severe disease. Finally, CCP also revealed a greater level of ventilation inhomogeneity (irrespective of severity of acute disease) in patients who had had COVID-19 compared with control participants, which was broadly the equivalent of that associated with 15 yr of lung aging. The absence of parameter values before infection means it is not possible to discern whether these findings form a set of lung-related risk factors for developing more severe disease or whether they are the direct result of infection with COVID-19 and/or its management.

## GRANTS

This work was supported by the National Institute for Health Research (NIHR) Oxford Biomedical Research Centre (BRC). The MCOVID project was funded by the Defence Medical Services Group. The C-MORE-LP project was supported by a grant awarded to N.P. and N.P.T. from the University of Oxford COVID-19 Research Response Fund. S.R.M.M.-E. was supported by a British Heart Foundation Doctoral Training Centre studentship at the University of Oxford. A.A. was supported by a scholarship from the Saudi Government. D.S. was supported by a Clarendon Scholarship from the University of Oxford. B.R. was supported by an Oxford British Heart Foundation (BHF) Centre for Research Excellence (CRE) fellowship (RE/18/3/34214). N.P. was supported by a NIHR research capability fund (RCF) award from the Oxford University Hospitals.

## DISCLAIMERS

The views expressed are those of the authors and not necessarily those of the National Health Service (NHS), the NIHR, or the Department of Health.

## DISCLOSURES

Oxford University Innovation, a wholly owned subsidiary of the University of Oxford, holds/has filed patents relating to the background IP for the technology. J.H.C., G.A.D.R., and P.A.R. have an interest in one or more patents. B.R. has consulted for Axcella therapeutics. None of the other authors has any conflicts of interest, financial or otherwise, to disclose.

## AUTHOR CONTRIBUTIONS

P.A.R. and G.A.D.R. conceived the study and technology for computed cardiopulmonography; D.A.H., E.D.N., and A.N.B. conceived, planned, and organized the MCOVID cohort study; M.P.C., N.P., B.R., and N.P.T. conceived, planned, and organized the C-MORE cohort study; B.R. planned and organized lung function testing; E.S. and C.X. organized and interpreted radiology; S.R.M.M.-E., A.A., R.R.C., H.X., R.P.McD., D.O.’B., B.C, J.C., and J.W. conducted experiments; D.S., C.J.F., and S.R.M.M.-E. developed software; J.H.C., N.M.J.S., and G.R. supported and developed the molecular flow sensor measurements; S.R.M.M.-E., A.A., T.W., N.P., and P.A.R. analysed data; S.R.M.M.-E., N.P., G.A.D.R., and P.A.R. interpreted results of experiments; S.R.M.M.-E., D.S., and N.M.J.S. prepared figures; P.A.R. drafted manuscript; S.R.M.M.-E., D.A.H., N.P., G.A.D.R., P.A.R., and N.P.T. edited and revised manuscript; S.R.M.M.-E., A.A., R.R.C., H.X., T.W., R.P.M., D.O., J.C., B.C., J.W., E.S., C.X., D.S., C.J.F., J.H.C., N.M.J.S., G.R., M.P.C., B.R., N.P.T., A.N.B., E.D.N., G.A.D.R., N.P., D.A.H., and P.A.R. approved final version of manuscript.

## APPENDIX

This appendix contains tables for the values for the computed cardiopulmonography parameters for the MCOVID cohort (Table A1) and for the C-MORE-LP cohort (Table A2). It also contains a table for the blood gas results for the MCOVID cohort together with related values predicted from the computational model (Table A3).

**Table A1.** MCOVID: computed cardiopulmonography parameters

	Control	Community	Ward	ICU	Overall
WHO severity	0	1–3	4–6	7–9	0–9
Fitted parameters					
Vd/ liters (BTPS)					
1st visit	0.133 ± 0.032	0.129 ± 0.034	0.125 ± 0.029	0.156 ± 0.060	0.131 ± 0.035
2nd visit	0.138 ± 0.042	0.135 ± 0.039	0.120 ± 0.027	0.163 ± 0.056	0.134 ± 0.039
δsVd					
1st visit	0.67 ± 0.33	0.49 ± 0.34***	0.51 ± 0.35	0.55 ± 0.32	0.54 ± 0.34
2nd visit	0.76 ± 0.38	0.54 ± 0.34	0.64 ± 0.30	0.56 ± 0.43	0.60 ± 0.35
Vdi/ liters (BTPS)					
1st visit	0.150 ± 0.033	0.143 ± 0.036	0.142 ± 0.033	0.179 ± 0.066*	0.147 ± 0.038
2nd visit	0.156 ± 0.045	0.151 ± 0.042	0.141 ± 0.027	0.191 ± 0.056	0.152 ± 0.041
Va/ liters (BTPS)					
1st visit	3.34 ± 0.84	2.97 ± 0.92	2.51 ± 0.76*	2.22 ± 0.41*	2.88 ± 0.89
2nd visit	3.73 ± 1.09	3.12 ± 0.97	2.37 ± 0.68	2.19 ± 0.35	2.96 ± 1.00
FRC/ % predicted					
1st visit	104 ± 23	91 ± 23	78 ± 20*	71 ± 15*	89 ± 24
2nd visit	117 ± 28	95 ± 24	73 ± 17	69 ± 13	91 ± 27
σVd					
1st visit	0.36 ± 0.06	0.37 ± 0.07	0.37 ± 0.09	0.33 ± 0.08	0.37 ± 0.08
2nd visit	0.30 ± 0.06	0.37 ± 0.06	0.33 ± 0.05	0.29 ± 0.06	0.35 ± 0.07
σlnCl					
1st visit	0.51 ± 0.08	0.55 ± 0.10**	0.59 ± 0.10**	0.57 ± 0.09	0.55 ± 0.10
2nd visit	0.50 ± 0.07	0.56 ± 0.10	0.57 ± 0.09	0.59 ± 0.11	0.56 ± 0.09
σlnCd					
1st visit	1.04 ± 0.25	1.10 ± 0.33	1.21 ± 0.32	1.00 ± 0.21	1.11 ± 0.31
2nd visit	1.12 ± 0.24	1.20 ± 0.29	1.11 ± 0.32	1.12 ± 0.34	1.16 ± 0.29
Pico_2_/ kPa					
1st visit	5.15 ± 0.46	5.16 ± 0.55	5.15 ± 0.61	5.26 ± 0.49	5.16 ± 0.54
2nd visit	5.30 ± 0.47	5.20 ± 0.58	5.32 ± 0.41	5.35 ± 0.42	5.26 ± 0.51
Pio_2_/ kPa					
1st visit	13.57 ± 1.03	13.80 ± 0.98	13.52 ± 1.18	13.59 ± 0.69	13.66 ± 1.02
2nd visit	13.63 ± 0.87	13.81 ± 1.00	13.33 ± 0.91	13.66 ± 0.98	13.65 ± 0.96
V̇o_2_/ liters (STPD)·min^−1^					
1st visit	0.330 ± 0.048	0.344 ± 0.061	0.319 ± 0.052***	0.344 ± 0.051	0.335 ± 0.056
2nd visit	0.324 ± 0.050	0.341 ± 0.053	0.333 ± 0.056	0.365 ± 0.042	0.338 ± 0.053
R					
1st visit	0.82 ± 0.08	0.85 ± 0.09	0.80 ± 0.09	0.82 ± 0.07	0.83 ± 0.09
2nd visit	0.84 ± 0.09	0.83 ± 0.08	0.78 ± 0.07	0.83 ± 0.06	0.82 ± 0.08
RER					
1st visit	0.90 ± 0.16	1.02 ± 0.19**	0.92 ± 0.16	0.90 ± 0.09	0.96 ± 0.18
2nd visit	0.86 ± 0.10	0.93 ± 0.14	0.84 ± 0.08	0.83 ± 0.07	0.89 ± 0.13

Values are means ± SD. Abbreviations for parameters estimated directly in the model are as defined in Table 1. ICU, intensive care unit; Pio_2_, partial pressure for O_2_ at the ideal point; RER, respiratory exchange ratio, as calculated directly from the data for the air-breathing period of the protocol; WHO, World Health Organization. **P* < 0.05, ***P* < 0.01, ****P* < 0.005.

**Table A2.** C-MORE-LP: computed cardiopulmonography parameter table

	Basic/O_2_	HFNC/CPAP	IMV	Overall
WHO severity	4–5	6	7–9	4–9
Fitted parameters				
Vd/ liters (BTPS)				
1st visit	0.133 ± 0.576	0.141 ± 0.577	0.152 ± 0.583**	0.140 ± 0.351
2nd visit	0.152 ± 0.577	0.136 ± 0.577	0.120 ± 0.584	0.140 ± 0.355
δsVd				
1st visit	0.894 ± 0.581	0.892 ± 0.580	0.833 ± 0.585	0.880 ± 0.473
2nd visit	0.941 ± 0.580	0.884 ± 0.579	0.818 ± 0.585	0.897 ± 0.472
Vdi/ liters (BTPS)				
1st visit	0.161 ± 0.572	0.172 ± 0.572	0.190 ± 0.579***	0.172 ± 0.348
2nd visit	0.181 ± 0.572	0.166 ± 0.573	0.162 ± 0.579	0.171 ± 0.352
Va/ liters (BTPS)				
1st visit	2.765 ± 1.197	2.640 ± 1.195	2.087 ± 1.196	2.564 ± 1.343
2nd visit	3.365 ± 1.195	2.626 ± 1.193	1.373 ± 1.194	2.756 ± 1.339
FRC/ % predicted				
1st visit	0.86 ± 0.56	0.80 ± 0.56	0.66 ± 0.57*	0.79 ± 0.44
2nd visit	0.96 ± 0.56	0.78 ± 0.56	0.54 ± 0.57	0.82 ± 0.44
σVd				
1st visit	0.39 ± 0.55	0.37 ± 0.55	0.41 ± 0.55	0.39 ± 0.34
2nd visit	0.35 ± 0.55	0.33 ± 0.55	0.40 ± 0.55	0.35 ± 0.34
σlnCl				
1st visit	0.63 ± 0.55	0.63 ± 0.55	0.70 ± 0.55	0.65 ± 0.39
2nd visit	0.63 ± 0.55	0.61 ± 0.55	0.80 ± 0.55	0.64 ± 0.39
σlnCd				
1st visit	1.23 ± 0.66	1.28 ± 0.66	1.36 ± 0.66	1.28 ± 0.62
2nd visit	1.29 ± 0.66	1.19 ± 0.66	1.21 ± 0.66	1.23 ± 0.62
Pico_2_/ kPa				
1st visit	5.02 ± 2.16	5.16 ± 2.16	5.15 ± 2.16	5.11 ± 2.44
2nd visit	5.23 ± 2.16	5.04 ± 2.16	4.69 ± 2.16	5.07 ± 2.44
Pio_2_/ kPa				
1st visit	13.82 ± 6.08	13.84 ± 6.08	13.41 ± 6.08	13.73 ± 6.77
2nd visit	13.55 ± 6.08	13.99 ± 6.07	14.43 ± 6.07	13.88 ± 6.76
V̇o_2_/ liters (STPD)·min^−1^				
1st visit	0.286 ± 0.560	0.296 ± 0.560	0.301 ± 0.567	0.294 ± 0.345
2nd visit	0.323 ± 0.560	0.315 ± 0.560	0.276 ± 0.567	0.314 ± 0.348
R				
1st visit	0.81 ± 0.56	0.82 ± 0.56	0.78 ± 0.56	0.81 ± 0.42
2nd visit	0.79 ± 0.56	0.83 ± 0.55	0.83 ± 0.56	0.81 ± 0.42
RER				
1st visit	0.93 ± 0.57	0.90 ± 0.57	0.92 ± 0.57	0.92 ± 0.46
2nd visit	0.87 ± 0.57	0.87 ± 0.57	0.83 ± 0.57	0.87 ± 0.46

Values are means ± SD. Abbreviations for parameters estimated directly in the model are as defined in Table 1. Basic/O_2_, standard hospital care with or without simple oxygen therapy; HFNC/CPAP, high-flow nasal cannula or continuous positive airway pressure; IMV, invasive mechanical ventilation; Pio_2_, partial pressure for O_2_ at the ideal point; RER, respiratory exchange ratio, as calculated directly from the data for the air-breathing period of the protocol; WHO, World Health Organization. **P* < 0.05, ***P* < 0.01, ****P* < 0.005.

**Table A3.** MCOVID: blood gas data and model predictions

	Control	Community	Ward	ICU	Overall
WHO severity	0	1–3	4–6	7–9	0–9
Blood parameters					
$\text{P}{\text{a}}_{\text{C}{\text{O}}_{2}}$/ kPa					
1st visit	4.89 ± 0.45	4.79 ± 0.60	4.87 ± 0.54	5.03 ± 0.15	4.84 ± 0.53
2nd visit	5.12 ± 0.43	4.79 ± 0.61	5.06 ± 0.40	5.30 ± 0.03	4.94 ± 0.52
$\text{P}{\text{a}}_{{\text{O}}_{2}}$/ kPa					
1st visit	13.83 ± 1.07	14.15 ± 1.27	13.57 ± 1.12	12.94 ± 0.96*	13.85 ± 1.22
2nd visit	14.49 ± 2.07	14.33 ± 1.40	13.03 ± 1.18	12.55 ± 0.35	13.92 ± 1.58
${\text{P}}^{\text{m}}{\text{a}}_{\text{C}{\text{O}}_{2}}$/ kPa					
1st visit	4.90 ± 0.58	4.72 ± 0.58	4.88 ± 0.43	4.86 ± 0.29	4.80 ± 0.53
2nd visit	4.97 ± 0.49	4.87 ± 0.69	5.15 ± 0.30	5.50 ± 0.73	5.00 ± 0.58
${\text{P}}^{\text{m}}{\text{a}}_{{\text{O}}_{2}}$/ kPa					
1st visit	13.29 ± 1.92	13.71 ± 1.51	12.62 ± 1.48	13.39 ± 0.51	13.35 ± 1.57
2nd visit	13.70 ± 1.45	13.48 ± 1.99	12.30 ± 1.64	11.74 ± 3.56	13.12 ± 1.91
($\text{P}{\text{a}}_{\text{C}{\text{O}}_{2}}$ − ${\text{P}}^{\text{m}}{\text{a}}_{\text{C}{\text{O}}_{2}}$)/ kPa					
1st visit	−0.01 ± 0.35	0.07 ± 0.38	−0.01 ± 0.34	0.18 ± 0.28	0.04 ± 0.36
2nd visit	0.14 ± 0.23	−0.10 ± 0.50	−0.10 ± 0.31	−0.20 ± 0.76	−0.06 ± 0.42
(${\text{P}}^{\text{m}}{\text{a}}_{{\text{O}}_{2}}$ − $\text{P}{\text{a}}_{{\text{O}}_{2}}$)/ kPa					
1st visit	−0.54 ± 1.20	−0.44 ± 1.39	−0.95 ± 1.57	0.44 ± 0.75	−0.51 ± 1.39
2nd visit	−0.79 ± 1.52	−0.84 ± 1.47	−0.73 ± 1.46	−0.81 ± 3.91	−0.80 ± 1.53
(iA–a)Po_2_/ kPa					
1st visit	0.71 ± 0.70	0.83 ± 1.01	0.85 ± 0.93	1.48 ± 0.71*	0.87 ± 0.93
2nd visit	0.42 ± 1.29	0.98 ± 0.89	1.15 ± 0.87	1.36 ± 2.52	0.93 ± 1.05
Δ$\text{P}{\text{a}}_{\text{C}{\text{O}}_{2}}$/ kPa					
1st visit	0.00 ± 0.33	0.08 ± 0.36	0.01 ± 0.33	0.16 ± 0.27	0.05 ± 0.34
2nd visit	0.15 ± 0.22	−0.08 ± 0.47	−0.08 ± 0.28	−0.16 ± 0.63	−0.04 ± 0.39

Values are means ± SD. ICU, intensive care unit; $\text{P}{\text{a}}_{\text{C}{\text{O}}_{2}}$, arterial CO_2_ partial pressure; $\text{P}{\text{a}}_{{\text{O}}_{2}}$, arterial O_2_ partial pressure; ${\text{P}}^{\text{m}}{\text{a}}_{\text{C}{\text{O}}_{2}}$, model estimate for the arterial CO_2_ partial pressure; ${\text{P}}^{\text{m}}{\text{a}}_{{\text{O}}_{2}}$, model estimate for the arterial O_2_ partial pressure; (iA–a)Po_2_, ideal alveolar to arterial oxygen gradient; $\Delta \text{P}{\text{a}}_{\text{C}{\text{O}}_{2}}$, the CO_2_ component of the orthogonal distance between the blood R line defined by the model and the measured arterial blood sample; WHO, World Health Organization. **P* < 0.05.
